# Performance of passive case detection for malaria surveillance: results from nine countries in Mesoamerica and the Dominican Republic

**DOI:** 10.1186/s12936-021-03645-x

**Published:** 2021-04-30

**Authors:** Diego Rios-Zertuche, Keith H. Carter, Katie Panhorst Harris, Max Thom, Maria Paola Zúñiga-Brenes, Pedro Bernal-Lara, Álvaro González-Marmol, Casey K. Johanns, Bernardo Hernández, Erin Palmisano, Rebecca Cogen, Paulami Naik, Charbel El Bcheraoui, David L. Smith, Ali H. Mokdad, Emma Iriarte

**Affiliations:** 1 Regional Malaria Elimination Initiative, Inter-American Development Bank, DC Washington, USA; 2 Institute for Health Metrics and Evaluation, University of Washington, Seattle, WA USA; 3 Regional Malaria Elimination Initiative, Inter-American Development Bank, San Jose, Costa Rica; 4 Regional Malaria Elimination Initiative, Inter-American Development Bank, Panama City, Panama; 5 Robert Koch Institute, Berlin, Germany

**Keywords:** Passive case detection, ABER, Annual blood examination rate, Malaria surveillance, Malaria elimination, Malaria indicators, Suspected malaria cases, Disease surveillance, Malaria detection, Malaria case positivity rate

## Abstract

**Background:**

In malaria elimination settings, available metrics for malaria surveillance have been insufficient to measure the performance of passive case detection adequately. An indicator for malaria suspected cases with malaria test (MSCT) is proposed to measure the rate of testing on persons presenting to health facilities who satisfy the definition of a suspected malaria case. This metric does not rely on prior knowledge of fever prevalence, seasonality, or external denominators, and can be used to compare detection rates in suspected cases within and between countries, including across settings with different levels of transmission.

**Methods:**

To compute the MSCT, an operational definition for suspected malaria cases was established, including clinical and epidemiological criteria. In general, suspected cases included: (1) persons with fever detected in areas with active malaria transmission; (2) persons with fever identified in areas with no active transmission and travel history to, or residence in areas with active transmission (either national or international); and (3) persons presenting with fever, chills and sweating from any area. Data was collected from 9 countries: Belize, Colombia (in areas with active transmission), Costa Rica, Dominican Republic, El Salvador, Guatemala, Honduras, Nicaragua, and Panama (September–March 2020). A sample of eligible medical records for 2018 was selected from a sample of health facilities in each country. An algorithm was constructed to assess if a malaria test was ordered or performed for cases that met the suspected case definition.

**Results:**

A sample of 5873 suspected malaria cases was obtained from 239 health facilities. Except for Nicaragua and Colombia, malaria tests were requested in less than 10% of all cases. More cases were tested in areas with active transmission than areas without cases. Travel history was not systematically recorded in any country.

**Conclusions:**

A statistically comparable, replicable, and standardized metric was proposed to measure suspected malaria cases with a test (microscopy or rapid diagnostic test) that enables assessing the performance of passive case detection. Cross-country findings have important implications for malaria and infectious disease surveillance, which should be promptly addressed as countries progress towards malaria elimination. Local and easy-to-implement tools could be implemented to assess and improve passive case detection.

## Background

Passive case detection (PCD) is perhaps the most critical element for malaria surveillance in all transmission settings, and it is particularly relevant in elimination settings to prevent re-establishing transmission in disease-free areas [1]. PCD encompasses the detection of malaria cases in people who seek care, usually with symptoms, from health providers or community health workers [1, 2]. The key challenge when an ill person presents to a health facility is determining whether they meet the criteria of a suspected malaria case and if a malaria test should be performed [2]. Medical staff first need to think of malaria and then determine if the clinical and epidemiological characteristics meet the case definition for malaria suspicion [2]. Malaria detection is the trigger for case investigations and active case detection [2]. In low transmission settings, misdiagnoses due to a lack of malaria suspicion have been shown to increase the risk of complications and death [3] and are also a potential route for re-introduction of imported cases [4].

It is estimated that the Region of the Americas has the highest potential for the elimination of *Plasmodium falciparum* and a high potential for *Plasmodium vivax *[5]. Most countries in Mesoamerica and the Dominican Republic are close to achieving malaria elimination. In 2018, the malaria incidence in Belize, Costa Rica, Dominican Republic, El Salvador, Guatemala, Honduras, Mexico, Panama, and Colombian municipalities bordering Panama was under 1 per 1000 population at risk, and, in Nicaragua, the incidence was below 10 per 1000 concentrated in a few municipalities [6]. Costa Rica reported zero autochthonous cases in 2013–2015, but 70 cases were reported in 2018. El Salvador has reported zero autochthonous cases since 2017 [6]. Over 93% of all infections reported in Mesoamerica are *P. vivax *[7].

Current malaria surveillance metrics have been insufficient to measure the performance of PCD adequately. For effective malaria surveillance, it is necessary to ensure that providers at all health facilities in the country suspect malaria when a case is presented [2]. The annual blood examination rate (ABER) has been used traditionally in areas with seasonal transmission [1, 8]. In theory, the ABER reflects the proportion of the at-risk population who met the case definition for malaria suspicion and were tested. The ABER considers screening the entire population of a malaria area, and blood examination of all fever cases and any other cases suspicious of having malaria. Passive and active case detection activities were considered adequate by the World Health Organization (WHO) if the proportion of blood slides examined during the previous 12 months reached a threshold [9]. Initially, if this rate exceeded 10%, case detection would be quantitatively adequate in all cases—currently, a specific threshold is not recommended [1]. In areas with seasonal transmission or with other epidemiological conditions, an ABER of 3–5% could be adequate, especially when determining criteria for discontinuation of spraying operations or for planning the number of microscopists needed [9]. However, the ABER has limitations: tests performed do not necessarily imply testing was conducted on suspected malaria cases—if a testing target is set, it could create perverse incentives to overreport; ABER has to be risk-adjusted for malaria transmission and population size, which complicates subnational comparisons; its interpretation relies on the assumption or knowledge of the baseline febrile illness for a given population and its seasonal variations, and it does not account for changes in the burden of other febrile illnesses. A more accurate metric reflecting how well patients are managed in health facilities is needed [10].

In the context of the Regional Malaria Elimination Initiative (RMEI), a metric was needed to verify country performance in PCD. RMEI seeks to eliminate malaria from ten countries in Mesoamerica and the Dominican Republic through a results-based financing model (RBF) and a collective impact framework. The RBF model entails national-level targets across indicators for all components for elimination [11]. If agreed upon targets are met, the country receives an award. Targets are verified externally for each of the two phases through health facility surveys, including a medical record review module, and population measurements.

For this purpose, a metric was developed to measure malaria tests on cases presenting to health facilities that meet a suspected malaria case definition. This metric, the suspected malaria cases with test (SMCT), can be used to measure the PCD performance across health facilities and compare PCD performance within and between countries, including across settings with different levels of transmission. Methods are based on previous experience measuring healthcare quality through medical record reviews in which criteria are transformed from check-lists into data points and decisions into conditional algorithms [12]. This document describes the methods used to measure the SMCT indicator. First, an explanation of the operational definition of suspected malaria is provided. Then, a description of the sample selection process and how the suspected case definition is incorporated into an algorithm to identify which cases meet the case definition and should have been tested. Finally, the manuscript summarizes how the data was collected and presents results for a baseline assessment in nine countries in Mesoamerica and the Dominican Republic.

## Methods

### Operational definition for suspected malaria cases

An operational definition of suspected malaria cases was adopted considering a combination of clinical and epidemiological characteristics. Fever is the most common clinical manifestation of malaria, especially in low-transmission settings [3, 10, 13, 14]. Hence, presenting to health facilities with current fever or recent fever history was an initial consideration for malaria suspicion [1, 2]. Fever history is essential to account for the usual progression of the disease with intermittent symptoms [15] as well as the intake of fever-reducing medications. Nevertheless, febrile illness is not specific to malaria; many other conditions have a similar clinical presentation. In addition to fever, sweating and chills are other symptoms that complete the typical triad for malaria, especially for *P. vivax *[16].

Given that these symptoms are not always present [13] establishing an epidemiological link to account for malaria risk was needed [2]. The risk of infection is mostly related to malaria transmission status in a particular area [1]. In areas without active malaria transmission, the malaria risk is almost exclusively related to travel from areas with active transmission. In most countries, municipal-level malaria risk stratification was conducted by the RMEI partnership considering the number of malaria cases, receptivity (mosquito presence), and vulnerability (population movement). In Costa Rica, Belize, and El Salvador, locality-level stratification was conducted, and health facilities classified according to the highest strata found in the catchment area. Municipalities were grouped into four strata: stratum 1, not receptive and not vulnerable; stratum 2, receptive but not vulnerable; stratum 3, receptive and vulnerable without autochthonous cases in the past three years; and stratum 4, active transmission. For strata 1–3, to account for malaria risk, the suspected case definition required travel history to stratum 4 or residence in stratum 4 (either sub-nationally or internationally) in addition to fever in the suspected case definition. In strata 4, patients presenting with fever alone merit malaria suspicion. Nevertheless, if someone in strata 1–3 presents with fever, sweating, and chills, malaria should also be suspected.

In summary, the basic definition for suspected malaria cases includes: (1) persons presenting with fever (or history of recent fever) detected in stratum 4; (2) persons presenting with fever (or history of recent fever) detected in stratum 1–3 who live in stratum 4 or with travel history to stratum 4 (or country with active transmission); and (3) persons presenting with fever (or history of recent fever), chills and sweating from any area (either concurrently or in sequence, at least once). History of recent fever is considered as any note in the medical record indicating fever in the days preceding the visit.

Although this definition may be desired in some settings, its specificity was increased by excluding cases of febrile illness with a defined aetiology according to the most frequent causes in each country. For example, excluding acute respiratory infections, skin lesions, urinary infections, and arboviruses with positive viral tests (any type of test noted on the medical record). Although cases of mixed infections are not uncommon, in many circumstances, the clinician’s judgment is needed to decide whether a malaria test is carried out. This exclusion ensures that the suspected case definition focuses on cases where a malaria test should have been performed without a doubt. Similar approaches have been recommended for settings with low endemicity [17]. Besides, this suspected malaria case definition is intended for measurement purposes and is not meant to guide clinical practice.

Finally, signs and symptoms that have been known to be present in malaria cases without febrile illness [13] were incorporated: splenomegaly, hepatomegaly, jaundice, and headache. These diagnoses are only considered for areas with active malaria transmission, encompassed by stratum 4, where malarial infections could have caused the illnesses.

In most countries, the definition used is more specific than the suspected malaria case definition in the country’s national malaria norms or guidelines in 2018. Table 1 summarizes the suspected case definition valid in each country for this period.

**Table 1:** Suspected case definitions on country malaria norms and guidelines valid in 2018 Sources: Belize [18], Colombia[19], Costa Rica [20], Dominican Republic [21], El Salvador [22, 23], Guatemala [24], Honduras [25], Nicaragua [26], Panama [27]

Country	Patient with intermittent fever, chills, sweating	Current fever or recent fever (only)	Current or recent fever & Living in an endemic area	Current or recent fever & Travel to endemic area	Other symptoms	Other epidemiological factors
Belize	No	Yes, no time period	No	Yes, no period	Haemorrhagic manifestation, headache, vomiting, retro-orbital pain, arthralgia, myalgia, nausea	Contact with malaria case History of medication with antimalarials
Colombia	Yes	Yes, last two weeks	Yes, last 15 days	Yes, last 15 days	Headache, gastrointestinal symptoms, myalgia, arthralgia, nausea, vomit, anaemia, splenomegaly	History of blood transfusions or transplants in last 30 days Contact with malaria case History of malaria History of medication with antimalarials
Costa Rica	Yes	No	Yes, last 40 days	Yes, last 40 days	Headache, muscle pain	No
Dominican Republic	Yes	Yes, last two weeks	Yes, last 30 days	Yes, last 30 days	Headache, photophobia, muscle pain, lack of appetite, nausea, vomit	History of blood transfusions or transplants in last 30 days Neonate from mother with malaria History of accidental contact with blood with malaria History of malaria
El Salvador	No	Yes, last two weeks or more than 30 days	Yes, no period	Yes, no period	No	No
Guatemala	Yes	No	Yes, last 30 days	Yes, last 7 days	Headache, general malaise	History of malaria
Honduras	Yes	No	Yes, no period	Yes, no period	Headache	No
Nicaragua	Yes	No	No	No	No	No
Panama	Fever and chills only	No	No	No	Headache, weakness, fatigue, abdominal discomfort, myalgia, arthralgia	No

### Sample selection

The sample was selected through a two-step process: first selecting a sample of health facilities, and then selecting a sample of medical records. A list of all public health facilities in the country was obtained to select the sample of health facilities. In the sample, facilities in strata 3 and 4 were prioritized to represent areas at higher risk for malaria transmission, and facilities with testing capacity were over-represented to collect data for other indicators, due to sample size limitations. An initial random sample was obtained from facilities that provide primary care services for malaria. For each selected primary health facility, the associated ancillary units from the reporting chain (notification units, laboratories, referral hospitals) was included up to a fixed sample size defined to balance budget considerations with statistical power for analysis. In El Salvador, which has been free of transmission for over three years, strata 1 and 2 were also selected (stratum 1 facilities were referral hospitals for facilities in other strata). Upon elimination, providers in health facilities are expected to take over suspicion and detection of malaria cases in all areas [2].

**Fig. 1:**
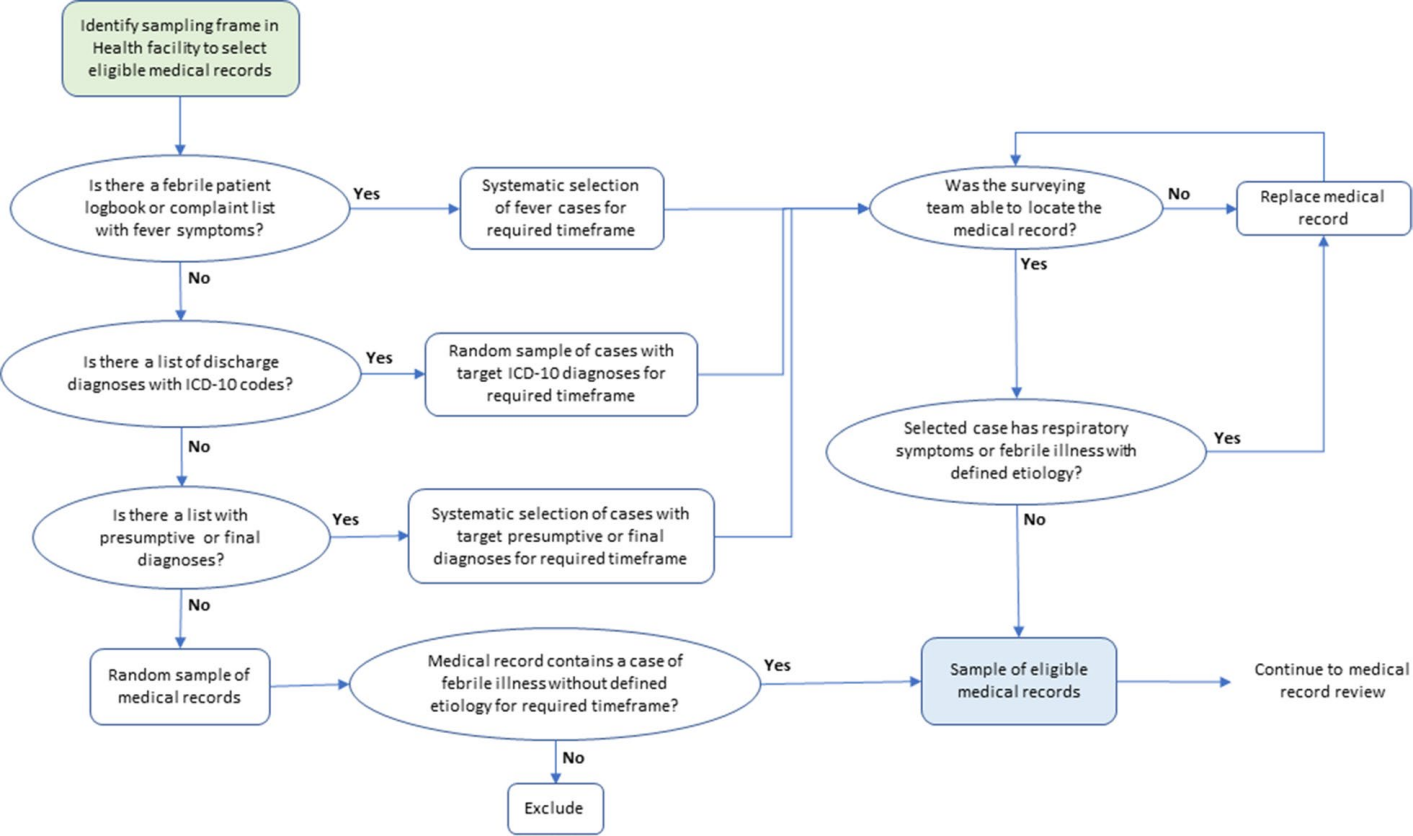
Medical record sample selection process

From each primary health facility or hospital, a sample of medical records was selected. Depending on the facility’s record-keeping system, a different sampling frame was used to select medical records: (1) if a book of fever cases or a complaint logbook was available, a systematic procedure was used to select records from the measurement timeframe, which included the possibility of selection for all suspected malaria cases in the calendar year of 2018 (this was the preferred sampling procedure); (2) if an electronic list of discharge records was available (usually with ICD-10 codes), a random sample from a predefined list of eligible diagnoses was selected for the measurement timeframe; (3) if a book with presumptive or final medical diagnoses was available, a systematic procedure was used to select cases from a predefined list of eligible diagnoses within the measurement timeframe. The list of diagnoses used to select records for each sampling frame are described in Additional file 1: Annex S1. If no sampling frame was available, a random sample of medical records was reviewed to find any fever encounters within the timeframe. If a medical record could not be found, it was replaced with another eligible record from a pre-selected list of substitutes. In all cases, the procedure entailed reviewing the medical record to check if exclusion criteria due to febrile illness with defined aetiology were present according to the suspected malaria case definition. Each of these ineligible records was replaced with an alternate record selected to a back-up randomly selected sample to ensure completion of the total quota for medical record reviews in each facility. In some primary care facilities, field personnel found an inadequate number of eligible attentions from the year 2018 to meet the quota, and all eligible cases from 2018 were reviewed. For medical records selected from presumptive diagnoses or discharge records, the procedure also entailed confirming the fever diagnosis when the target diagnosis did not necessarily imply fever (unless the health facility was in stratum 4 for signs and symptoms without febrile illness). The medical record selection process is summarized in Fig. 1.

### Algorithm for suspected malaria cases with parasitological test

**Fig. 2:**
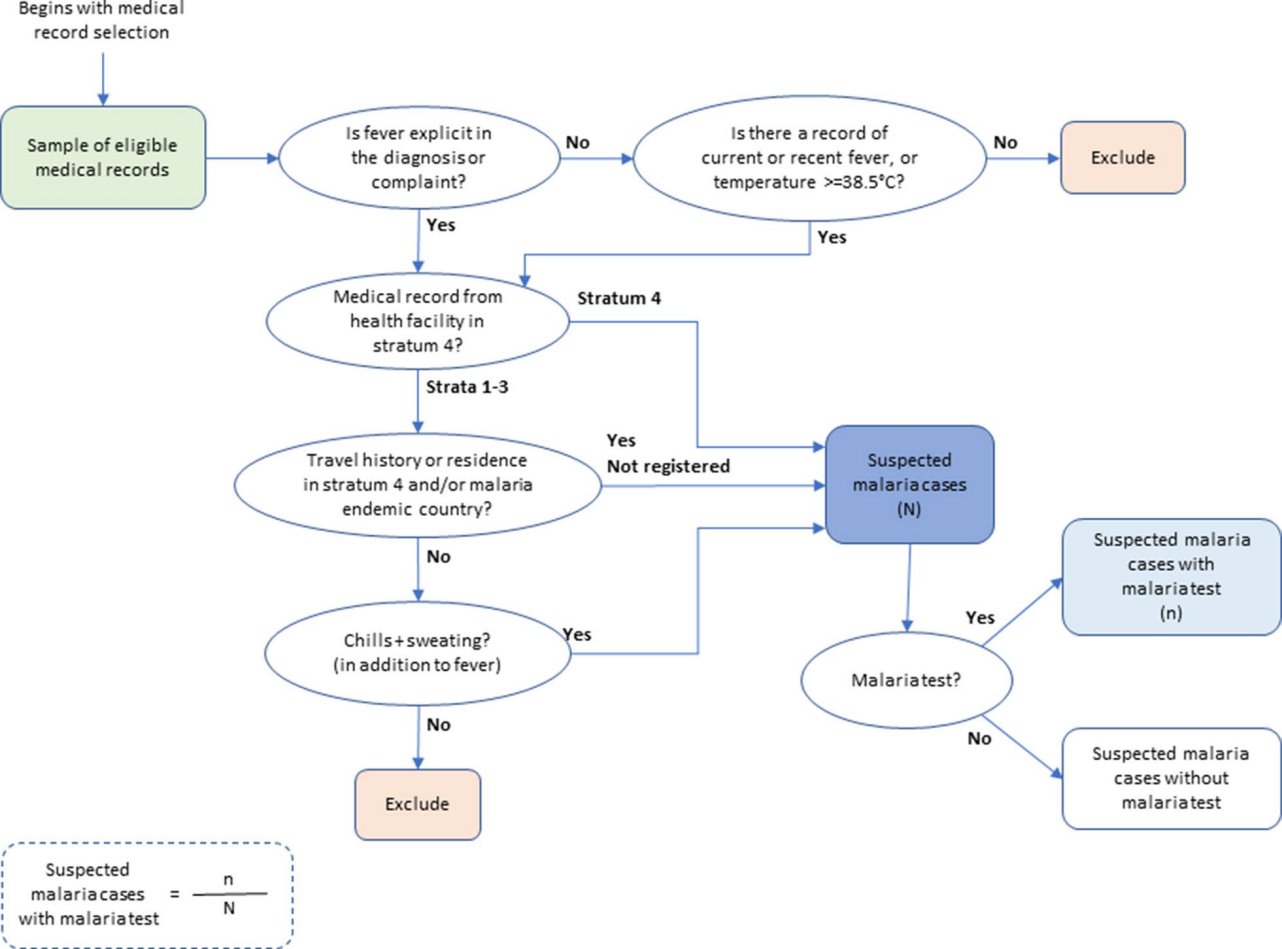
Algorithm for suspected malaria cases with malaria test

An algorithm was developed to assess if a malaria test was ordered for suspected malaria cases. The algorithm first checked if the medical record met the suspected malaria case definition (see Fig. 2). All medical records extracted from health facilities in stratum 4 were considered suspected malaria cases. For cases in strata 1–3, the algorithm first checked the patient’s travel history in the past 30 days, including migration, and place of residence. Patients with travel history or residence in stratum 4 were considered suspected malaria cases. If travel history or residence was not recorded, they were also considered suspected malaria cases – which is meant to encourage registration practices as part of RMEI’s RBF model and is part of the quality of the diagnosis. If no travel history nor residence in stratum 4 was found, the algorithm checked for the presence of chills and sweating in addition to fever. If chills and sweating were recorded, the patient was considered a suspected malaria case. Otherwise, the patient record was excluded.

Then, the algorithm checked if a parasitological test was ordered. If any indication was found that a microscopy test or rapid diagnostic test (RDT) for malaria was ordered or performed, the medical record was considered compliant. For example, a note stating that an RDT was performed, the positive or negative results of the test, a note stating that a thick blood smear for malaria was taken, laboratory results for malaria, and similar statements. The note could be present in any part of the medical record, and, in some countries, the patient’s name on logbooks or registries for malaria tests were also acceptable. If no record for malaria tests were found, the medical record was considered not compliant. The final calculation of the SMCT indicator considered the number of suspected malaria cases in the denominator and the number of suspected malaria cases with a malaria test in the numerator.

### Data collection

Data was collected from nine countries: Belize, Colombia, Costa Rica, Dominican Republic, El Salvador, Guatemala, Honduras, Nicaragua, and Panama. Data collection was performed by medical doctors and nurses from each country. Electronic chart extraction tools were designed to collect data from selected medical records using computer-assisted personal interviewing software (SurveyCTO). Built-in quality controls were incorporated, such as required responses, date checks, and real value checks. The surveying software rendered a de-identified database with no personal information. Data collection took place between September 2019–March 2020; however, in all countries, data was collected for fever cases that occurred in 2018. In Colombia, data collection, which began in February 2020, was suspended due to COVID-19 in March, which implied that the target sample size could not be reached.

## Results

A total of 6639 medical records were reviewed in all nine countries. Table 2 describes the number of medical records considered at each step of the study. 16.4% of the medical records initially selected could not be located. Of all records reviewed, 8.2% were discarded and replaced after an initial screening for eligibility. After reviewing all eligible medical records, 5873 records from 239 facilities were determined to be suspected malaria cases according to the definition.

**Table 2:** Study design for medical records selected for review

	Belize	Colombia	Costa Rica	Dominican Republic	El Salvador	Guatemala	Honduras	Nicaragua	Panama	Total
Total medical records selected for review^a^ (including replacements)	897	278	983	632	842	1269	1868	1233	645	8647
Selected medical records that could not be located for review^b^	39	11	120	122	33	8	935	139	7	1414
All medical records screened for eligibility^c^	858	267	863	510	809	1261	933	1094	638	7233
Ineligible medical records replaced^d^	7	13	19	44	196	33	81	128	72	593
Eligible medical records collected^e^ (starting point of algorithm)	851	254	843	466	613	1228	852	966	566	6639
Suspected malaria cases^f^ (denominator)	836	254	364	460	516	1196	801	891	555	5873

^a^Considers all medical records selected from the sampling frame (logbook, list of diagnoses or discharge records)

^b^Physical or electronic medical records that could not be located

^c^Medical records located, and screened

^d^Replaced after screening for eligible diagnosis or febrile illness of defined aetiology

^e^Medical records fully reviewed

^f^Medical records that met the definition of suspected malaria case

The majority of the medical records were reviewed in health facilities in strata 3 and 4. In most countries, malaria tests on persons who satisfied the definition of a suspected case were below 10% with the exceptions of Nicaragua and Colombia. shows the SMCT indicator results by country (see Table 3).

**Table 3:** Medical record review of suspected malaria cases, January–December 2018

Country	Health facilities		Suspected malaria cases with malaria test			
	Primary health facilities	Hospitals	N	n	%	95% CI
Belize	10	7	836	22	2.6	[1.7─4.0%]
Colombia^a^	9	2	254	98	38.6	[32.8─44.8%]
Costa Rica	19	4	364	2	0.6	[0.1─2.2%]
Dominican Republic	24	9	460	13	2.8	[1.6─4.8%]
El Salvador^b^	16	3	516	71	13.8	[11.0─17.0%]
Guatemala	38	5	1196	62	5.2	[4.1─6.6%]
Honduras^c^	32	5	801	67	8.4	[6.6─10.5%]
Nicaragua	33	4	891	723	81.1	[78.4─83.6%]
Panama	19	0	555	52	9.4	[7.2─12.1%]

Unless otherwise stated, data from health facilities in strata 3 and 4

*CI* confidence Interval

^a^Sample includes municipalities in stratum 4 in the departments of Buenaventura, Nariño, and Chocó; only 16.4% of the expected sample size was collected

^b^Includes data from health facilities in strata 1–4

^c^Majority of the data from stratum 4, only data from four health facilities in stratum 3 is included

Malaria suspicion was higher in stratum 4 compared to stratum 3 in all countries with the exceptions of the Dominican Republic and Belize. While in Belize malaria suspicion was equally low across strata 3 and 4, suspicion was higher in stratum 3 than 4 in the Dominican Republic. In Nicaragua, all patients with fever are routinely registered to a list of suspected malaria cases and tested in stratum 4, which may explain the high percentage of suspected cases who were tested. Table 4 summarizes results by strata. Nevertheless, in all countries, more than 90% of cases reviewed lacked registration of patient travel history, and thus qualified as suspected malaria cases.

**Table 4:** Suspected malaria cases with malaria tests by strata, January—December 2018

Country	% records with data recorded (strata 1–3)		Suspected malaria cases with malaria test (%)				
	Place of residence	travel history	Stratum 4	Stratum 3	Stratum 2	Stratum 1	Total
Belize	84.8	0.2	2.0	3.0			2.6
Colombia^a^			38.6				38.6
Costa Rica	97.9	0.8	1.6	0.0			0.6
Dominican Republic	83.2	0.0	0.4	5.8			2.8
El Salvador^b^	64.1	0.0	24.8	14.6	2.9	3.3	13.8
Guatemala	56.3	0.6	6.0	0.6			5.2
Honduras^c^	98.0	6.0	8.9	0.0			8.4
Nicaragua	84.5	2.3	91.6	10.4			81.1
Panama	68.3	8.5	18.3	2.8			9.4

^a^Sample includes municipalities in stratum 4 in the departments of Buenaventura, Nariño, and Chocó; only 16.4% of the expected sample size was collected

^b^Stratum 1 data collected in only two referral hospitals

^c^Stratum 3 data collected in only four health facilities

During the data collection process, relevant challenges for malaria detection across countries were also observed. Only Nicaragua had implemented guidelines to manage fever cases, although they were only strictly followed in fever management clinics. In most countries, poor record-keeping and medical record management practices were observed. In at least four countries, medical records from 2018 had been lost or destroyed (by preventable accidents and negligence) in several health facilities. Similarly, due to poor archiving practices, the data collection team was unable to locate selected medical records in some health facilities. For example, medical records were archived under the name of a family member, but presumably, only the patient knew the name used. A medical record, or any other record, was often not completed for foreigners who sought care in public health facilities. In some instances, the records located were empty.

## Discussion

A statistically comparable, replicable, and standardized metric to measure the performance of PCD was proposed. This method to measure suspected malaria cases with malaria test (SMCT) addresses many of the existing challenges to ensure malaria surveillance is adequately implemented. This experience has shown that the proposed method could be used at scale to compare performance within and between countries. Findings from countries in Mesoamerica and the Dominican Republic are relevant not only for malaria surveillance, but also for infectious disease surveillance in general. The study uncovered important challenges in healthcare provision and PCD that could hinder progress in these countries towards malaria elimination.

From a methodological perspective, the SMCT indicator offers several advantages for malaria elimination settings. First, the sampling process does not require prior knowledge of fever prevalence, malaria seasonality, or external denominators (such as population) [28]. The SMCT’s numerator and denominator are self-contained in the medical record sample. The same metric can be used to compare PCD performance within and across countries, as granular as health facilities, and as aggregated as the sampling method allows. The interpretation of the resulting value is equivalent across different geographic units, levels of aggregation and time periods. Second, the suspected malaria case definition can be adapted to represent different operational guidelines or clinical protocols. Criteria may be adjusted to make the suspected malaria case definition more stringent or lenient depending on the testing strategy adopted. The combination of clinical and epidemiological criteria supports monitoring targeted testing efforts, instead of calculating detection efforts based on high-level estimates. Given that the results are based on actual observed cases, it is possible to make stakeholders accountable—health providers for suspecting malaria, regional managers for tracking supplies for tests, national decision-makers for planning adequate staffing, between others. While setting a target for ABER has been disputed, and a specific level is no longer recommended [1, 28], it is possible to set a standard-based target for the SMCT indicator.

Measuring testing on suspected cases through medical record reviews also enables monitoring PCD performance locally. A small sample of medical records could be extracted periodically in health facilities to assess PCD practices. Similar methods have already been used by Ministries of Health in Mesoamerica to improve healthcare quality [12].

In the nine countries surveyed, the data collection process uncovered patient management and surveillance challenges. The lack of registration of recent travel history in all countries, and place of residence in some countries, is troubling. Travel history is a significant indication of malaria risk for areas without active transmission [3, 29]. Although questions around travel history are part of malaria case investigation forms, these forms are only completed once a case is confirmed [2]. Assessing the travel history to screen fever cases is not only inexpensive compared to testing, but is also a relevant intervention for infectious disease surveillance in general. The lack of fever management processes, record-keeping, medical record management, and archiving practices adds complexity to the already demanding task of disease surveillance. Most of these issues have ramifications in the quality of care provided to patients beyond malaria and should be promptly addressed.

The finding that testing was higher in strata with active malaria transmission is consistent with other studies. While malaria is top-of-mind for health staff in malaria-endemic areas, symptoms may be overlooked in other areas [3, 16]. In the Dominican Republic, increased testing in stratum 3 may be explained by the health system structure, where hospitals, located outside active malaria foci, have a more significant role in patient care compared to primary health facilities. Lessons from other diseases have shown that a clear case definition that is easily applicable by health workers is essential to identify cases early and prevent outbreaks [30]. In countries en-route to elimination, the suspected malaria case definition must have flexibility to adapt as the areas with active malaria transmission change.

At least two countries in Mesoamerica recently updated their suspected case definitions for malaria and passive case detection protocols [31, 32]. In El Salvador, the updated clinical guidelines include a detailed algorithm to support passive surveillance and testing decisions [31]. A clear definition for suspected malaria cases, job aide tools to screen fever cases and clear fever management processes should be implemented across countries pursuing malaria elimination [33]. Using metrics such as the SMCT indicator to monitor adherence with clinical guidelines will be critical to ensure country-wide compliance. Analysing the positivity rate and methods of diagnosis available could be useful to adjust the sensitivity of the malaria suspected case definition and diagnostic accessibility, which are important areas for future study.

The proposed metric also has limitations. A common critique of medical record reviews is that they measure documentation practices instead of actual care [12]. For instance, it is not possible to determine if health providers inquired about travel history and did not record their findings, or if they did not inquire at all. The only way to confirm that processes are carried out systematically is by requiring documentation. In many situations, documentation is critical to determine the course of action, such as for case investigations and treatment failures. Studies have found that documentation increases adherence to clinical practices and improves outcomes [34, 35]. If data collection instruments are properly designed, data collection for the SMCT exposes problems with data quality, adherence to standard operating procedures, and record keeping. The resulting data can be used to adjust estimates and is a valuable input for healthcare quality improvement.

The SMCT indicator, or its computational algorithm, should not substitute for clinical judgement. There are instances that merit malaria testing even if the suspected case definition is not met. Healthcare workers with experience are more likely to suspect from uncommon signs of disease [30], and should have flexibility to suspect malaria even after a negative malaria test result [31]. Furthermore, to be effective, the country’s malaria detection and diagnosis strategy must be strongly aligned with its case management and response strategy. Positive cases should receive prompt treatment and be rapidly investigated to trigger proactive case detection [11]. Considering that the sample represents areas and health facilities more likely to suspect and diagnose malaria, these findings underscore that strong surveillance, case management protocols, and guidelines are necessary but not sufficient. Ensuring guidelines are applied consistently by all health workers is crucial.

## Conclusions

Malaria PCD should be conducted by all health care professionals across all countries [2]. A new metric was proposed to measure passive case detection and compare detection rates in suspected cases within and between countries, including settings with different levels of transmission. The SMCT indicator could be measured locally to improve passive case detection rates. Study findings underscore the need for countries in Mesoamerica and the Dominican Republic to refine their suspected malaria case definitions, establish clear processes to screen for infectious diseases nationally, and implement interventions to strengthen malaria case detection. Improving medical record documentation and record-keeping practices is also necessary. Passive case detection is a critical link for malaria surveillance; ensuring its adequate performance is fundamental as countries continue progressing towards malaria elimination and to prevent the re-establishment of transmission.

## Supplementary Information

**Additional file 1.** List of diagnoses to select medical records.

## Data Availability

The datasets used and/or analysed during the current study are available from the corresponding author on reasonable request.

## Abbreviations

ABER: Annual blood examination rate
CI: Confidence interval
ICD-10: International Classification of Diseases Version 10
PCD: Passive case detection
RBF: Results-based financing
RDT: Rapid diagnostic test
RMEI: Regional malaria elimination initiative
SMCT: Suspected malaria cases with test
WHO: World Health Organization
