# Supplementary material for: Performance of passive case detection for malaria surveillance: results from nine countries in Mesoamerica and the Dominican Republic
Source: Malar J. 2021 Apr 30;20:208. doi: 10.1186/s12936-021-03645-x (PMC8085801; doi:10.1186/s12936-021-03645-x)
Supplement: Supplementary file 1 — Additional file 1. List of diagnoses to select medical records. [file 12936_2021_3645_MOESM1_ESM.pdf]

Rios-Zertuche et al. *Malar J*
https://doi.org/10.1186/s12936-021-03645-x

Malaria Journal
Supplementary File

Performance of passive case detection for malaria surveillance: results from nine countries in Mesoamerica and the Dominican Republic

Annex S1: List of Diagnoses to Select Medical Records

Contents

Diagnoses considered for medical record selection 1
Sampling by principal complaint - motives eligible for review 1
Sampling by presumptive or final diagnosis - diagnoses eligible for review 1
Sampling by ICD code - diagnoses eligible for review 1
Diagnoses ineligible unless fever was documented: 2
Sampling by ICD code – ineligible unless fever was documented in all health facilities 2
Sampling by presumptive or final diagnosis – ineligible unless fever was documented in all health facilities 3
Sampling by ICD code – ineligible unless fever was documented in health facilities of strata 1-3 3
Sampling by presumptive or final diagnosis – ineligible unless fever was documented in health facilities of strata 1-3 3
Diagnoses ineligible for record review (febrile illnesses with defined aetiology) 3

Diagnoses considered for medical record selection

Sampling by principal complaint - motives eligible for review

- Fever
- Malaria
- Dengue
- Chikungunya

Sampling by presumptive or final diagnosis - diagnoses eligible for review

- Fever (acute, relapsing, persistent, unspecified, etc.)
- Malaria (*P. falciparum*, *P. vivax* or unspecified)
- Leptospirosis
- Dengue (classical, haemorrhagic or unspecified)
- Chikungunya
- Mosquito-borne fever
- Viral infection, unspecified
- Meningitis
- Hepatomegaly
- Splenomegaly

Sampling by ICD code - diagnoses eligible for review

- A41.9 Sepsis, unspecified organism
- A68 Relapsing fevers
- A68.9 Relapsing fever, unspecified
- A98.5 Haemorrhagic fever with renal syndrome

- B34.9 Viral infection, unspecified
- B50 *Plasmodium falciparum* malaria
- B50.0 *Plasmodium falciparum* malaria with cerebral complications
- B50.8 Other severe and complicated *Plasmodium falciparum* malaria
- B50.9 *Plasmodium falciparum* malaria, unspecified
- B51 *Plasmodium vivax* malaria
- B51.0 *Plasmodium vivax* malaria with rupture of spleen
- B51.8 *Plasmodium vivax* malaria with other complications
- B51.9 *Plasmodium vivax* malaria without complication
- B52 *Plasmodium malariae* malaria
- B52.0 *Plasmodium malariae* malaria with nephropathy
- B52.8 *Plasmodium malariae* malaria with other complications
- B52.9 *Plasmodium malariae* malaria without complication
- B53 Other specified malaria
- B53.0 *Plasmodium ovale* malaria
- B53.1 Malaria due to simian plasmodia
- B53.8 Other malaria, not elsewhere classified
- B54.X Unspecified malaria
- G03.9 Meningitis, unspecified
- R16 Hepatomegaly and splenomegaly, not elsewhere classified
- R16.1 Splenomegaly, not elsewhere classified
- R16.2 Hepatomegaly with splenomegaly, not elsewhere classified
- R17.X Unspecified jaundice
- R50 Fever of other and unknown origin
- R50.0 Fever with chills
- R50.1 Persistent fever
- R50.8 Other specified fever
- R50.9 Fever, unspecified
- R51.X Headache
- R68 Other general symptoms and signs
- R68.8 Other general symptoms and signs
- A27 Leptospirosis
- A27.0 Leptospirosis enterohemorrhagic
- A278 Other forms of leptospirosis
- A279 Leptospirosis, unspecified
- A90.X Dengue fever [classical dengue]
- A91.X Dengue haemorrhagic fever
- A92 Other mosquito-borne viral fevers
- A92.0 Chikungunya virus disease
- A92.8 Other specified mosquito-borne viral fevers
- A92.9 Mosquito-borne viral fever, unspecified

Diagnoses ineligible unless fever was documented:

Sampling by ICD code – ineligible unless fever was documented in all health facilities

- A41.9 Sepsis, unspecified organism
- B34.9 Viral infection, unspecified
- G03.9 Meningitis, unspecified
- R68 Other general symptoms and signs
- R68.8 Other general symptoms and signs
- A27 Leptospirosis

- A27.0 Leptospirosis enterohemorrhagic
- A27.8 Other forms of leptospirosis
- A27.9 Leptospirosis, unspecified

Sampling by presumptive or final diagnosis – ineligible unless fever was documented in all health facilities

- Leptospirosis
- Viral infection, unspecified
- Meningitis

Sampling by ICD code – ineligible unless fever was documented in health facilities of strata 1-3

- R16 Hepatomegaly and splenomegaly, not elsewhere classified
- R16.1 Splenomegaly, not elsewhere classified
- R16.2 Hepatomegaly with splenomegaly, not elsewhere classified
- R17.X Unspecified jaundice
- R51X Headache

Sampling by presumptive or final diagnosis – ineligible unless fever was documented in health facilities of strata 1-3

- Hepatomegaly
- Splenomegaly

Diagnoses ineligible for record review (febrile illnesses with defined aetiology)

- Arbovirus with positive viral test
  - Dengue
  - Chikungunya
  - Zika
- Acute respiratory infection
- Gastrointestinal infection
- Fever of neurological origin
- Skin lesion
- Urinary infection
- Findings in soft tissues
- Focal infection
- Other parasitological infection
